# A systems-level machine learning approach uncovers therapeutic targets in clear cell renal cell carcinoma

**DOI:** 10.1038/s44386-025-00036-z

**Published:** 2026-02-09

**Authors:** Silas Ruhrberg Estévez, Greta Baltusyte, Gehad Youssef, Namshik Han

**Affiliations:** 1 Milner Therapeutics Institute, University of Cambridge, Cambridge, UK; 2 Cambridge Centre for AI in Medicine, University of Cambridge, Cambridge, UK; 3 Cambridge Stem Cell Institute, University of Cambridge, Cambridge, UK; 4 Department of Surgery, University of Cambridge, Cambridge, UK; 5 Urological Malignancies Virtual Institute, University of Cambridge, Cambridge, UK; 6 Department of Quantum Information, Institute for Convergence Science Academy, Yonsei University, Seoul, Republic of Korea; 7 Department of Nano Biomedical Engineering (NanoBME), Advanced Science Institute, Yonsei University, Seoul, Republic of Korea; 8 Center for Nanomedicine, Institute for Basic Science (IBS), Seoul, Republic of Korea

**Keywords:** Cancer, Computational biology and bioinformatics, Drug discovery

## Abstract

Clear cell renal cell carcinoma (ccRCC) is an aggressive malignancy with limited treatment options and high rates of resistance to first-line kinase inhibitors. Current therapies largely target the tumor microenvironment, leaving intrinsic tumor vulnerabilities underexplored. Here, we introduce a systems-based machine learning pipeline that integrates single-cell RNA sequencing, protein interaction networks, and drug proximity analysis to identify therapeutic targets in ccRCC. Candidate genes were refined using CRISPR screening data and functional relevance and validated across independent transcriptomic datasets. The pipeline recovered several established treatment pathways and uncovered previously underexplored therapeutic mechanisms, including ABL1, CDK4/6, and JAK inhibition. We identified FDA-approved compounds acting through these pathways, three of which, Ribociclib, Ponatinib, and Dasatinib, showed superior efficacy to current therapies across renal cancer cell lines in preclinical screens. By acting through mechanisms distinct from current therapies, they represent promising candidates for combination strategies aimed at overcoming resistance and improving clinical outcomes in ccRCC.

## Introduction

Each year, kidney cancer is diagnosed in 400,000 individuals worldwide, with its global incidence doubling over the past three decades^1,2^. Over 90% of kidney cancers originate from the renal epithelium, with clear cell renal cell carcinoma (ccRCC) being the most common subtype, accounting for ~75% of cases^3^. ccRCC is an aggressive malignancy with high metastatic potential and significant mortality^4^. For localised disease, partial or radical nephrectomy remains the standard of care and generally yields favorable outcomes^5^. However, around 20% of patients present with metastatic disease at diagnosis, and a further 30% of those who undergo surgery subsequently develop metastases^5^. Metastatic ccRCC poses a substantial clinical challenge due to its propensity to disseminate to multiple organ systems and its limited responsiveness to systemic therapies^6^. Despite recent advances, treatment options for advanced ccRCC remain inadequate, with a 5-year survival rate below 10%^5^. These poor outcomes, coupled with high rates of drug resistance, underscore the urgent need for novel therapeutic strategies^7^.

Currently, 15 FDA-approved drugs are available for the treatment of kidney cancer, classified into four groups^8^. Immunotherapies, including Ipilimumab, Avelumab, Pembrolizumab, Nivolumab, and Aldesleukin enhance the immune system’s ability to recognise and eliminate tumor cells by targeting immune checkpoints such as CTLA-4 and PD-L1, or by stimulating T-cell proliferation through the IL-2 pathway. Angiogenesis inhibitors, such as Bevacizumab, Axitinib, Belzutifan, Tivozanib, Sorafenib, and Sunitinib, impair tumor vascularization by interfering with VEGF or HIF-2α signaling pathways. The mTOR inhibitors Everolimus and Temsirolimus block tumor growth by inhibiting mTOR signaling. Multitarget kinase inhibitors, including Cabozantinib and Lenvatinib, represent a newer class of therapeutics to simultaneously inhibit VEGF receptors and other tumor-promoting pathways^3^.

Despite the availability of multiple therapeutic agents, treatment responses in ccRCC remain suboptimal. Resistance to targeted therapies typically emerges within 8–9 months of initiating first-line treatment, and within 5–6 months for second-line regimens^3^. Furthermore, many patients derive limited benefit from non-targeted therapies: the response rate for the immune checkpoint inhibitor Nivolumab is estimated at ~25%, while Everolimus shows a response rate of only 5%^9^. Traditional chemotherapy agents such Vinblastine or 5-fluorouracil are not commonly used given their response rates below 10% and their association with nephrotoxicity and renal injury^10,11^. Moreover, most currently approved drugs act by modulating the immune or vascular microenvironment rather than directly targeting tumor-intrinsic pathways.

Several efforts have attempted to address these limitations by screening for compounds that target molecular markers identified through genomic data^12,13^. Drug repurposing represents a potentially efficient route to therapy development, as safety profiles and mechanisms of action are already well established^14^. Previous studies aiming to repurpose drugs for clear cell renal cell carcinoma (ccRCC) have focused on identifying differentially expressed genes using bulk RNA sequencing, or on single drug targets identified through single-cell RNA sequencing (scRNA-seq). While these approaches have yielded valuable insights, they often lack validation in external datasets and fail to capture gene expression dynamics and regulatory interactions at the single-cell level. Moreover, such strategies frequently overlook the complexity of protein–protein interactions and broader systems-level processes that drive ccRCC progression.

We introduce a systems-level machine learning framework for drug target discovery that integrates single-cell transcriptomics, supervised feature selection, protein interaction networks, and drug proximity analysis. Applied to clear cell renal cell carcinoma (ccRCC), this pipeline identifies five therapeutic mechanisms not currently used in standard ccRCC care that demonstrate efficacy in preclinical validation. Unlike prior approaches that rely on bulk transcriptomic data or single-target analyses, our method leverages the growing availability of public scRNA-seq datasets to enable rapid in silico validation across independent cohorts. By incorporating cellular-resolution gene expression data within a systems-level framework, our approach provides a more dynamic and biologically relevant model of the regulatory mechanisms driving ccRCC progression. The pipeline not only identifies previously unexplored therapeutic mechanisms but also recapitulates established treatment targets, reinforcing its robustness. Although this study focuses on ccRCC, the pipeline is readily adaptable to other cancer types and is designed as a generalizable tool for AI-enabled drug repurposing.

## Results

### Drug target selection using machine learning

**Fig. 1:**
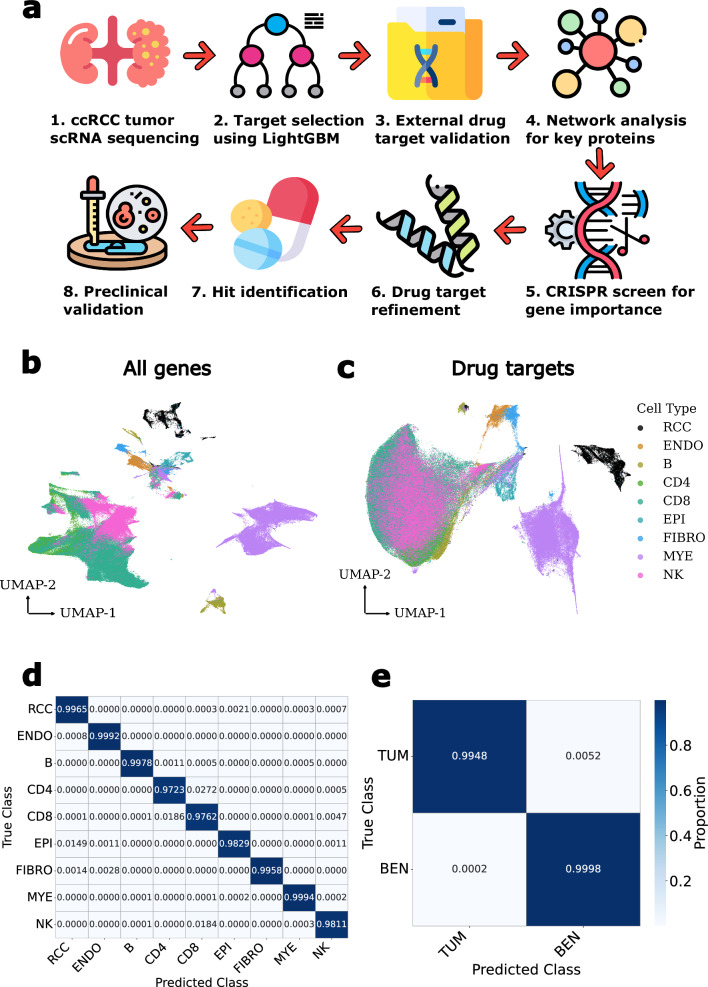
Integrative machine learning pipeline identifies tumor-specific gene signatures from single-cell RNA-seq data. **a** Overview of the systems-level drug discovery pipeline integrating single-cell RNA sequencing (scRNA-seq) with machine learning and network analysis. The pipeline employs LightGBM-based classification to identify tumor-intrinsic features, refines these using protein–protein interaction (PPI) network analysis to highlight key regulatory proteins, and applies drug–target proximity scoring to prioritize repurposable therapeutics for clear cell renal cell carcinoma (ccRCC). **b**-**c** UMAP visualizations of the training dataset showing cell type clusters (**b**) and tumor–normal separation based on the selected 96-gene feature subset (**c**), defined as genes with AUC > 0.8. **d** Confusion matrix of the multiclass classifier distinguishing nine major kidney cell types on validation data. **e** Confusion matrix of the binary classifier distinguishing tumor from non-tumor cells in validation data. Figure 1a was generated using icons from Freepik.

We developed a novel drug discovery pipeline that integrates single-cell RNA sequencing (scRNA-seq), machine learning, and network analysis (see Fig. 1a). The training dataset comprised over 270,000 cells collected from ten ccRCC tumors across multiple anatomical sites, as well as from adjacent healthy tissue samples^15^. In total, the dataset spans nine distinct cell types, as visualized in Fig. 1b, c.

To validate cell-type specificity, we trained both a multiclass LightGBM classifier to distinguish all cell types and a binary classifier to separate tumor from non-tumor cells^16^. Both models demonstrated high accuracy on held-out data (see Fig. 1d, e), indicating that the LightGBM architecture is well suited for capturing transcriptional differences in scRNA-seq data.

Next, we performed univariate feature selection with five-fold cross-validation to identify genes that best discriminated against tumor cells from normal cells. Using a threshold of area under the receiver operating characteristic curve (AUC) ≥ 0.8, we identified 96 genes as potential drug targets (see Supplementary Table 2 and 3). The distribution of AUC values for these genes is presented in Supplementary Fig. 1A. A full list of gene names and their corresponding AUC scores is available in Supplementary File 1, while log_2_ fold changes in expression between tumor and non-tumor cells are provided in Supplementary File 2.

To evaluate stability and cross-method consistency, we compared the univariate AUC-selected genes with three model-based attributes: Logistic Regression coefficients, LightGBM feature importance, and SHAP values. Before downstream filtering using the network analysis, the UFS set overlapped by 50% with LightGBM, 53% with SHAP, and 95% with Logistic Regression; after applying the methodological filters described in the Methods, overlap among the retained gene sets increased to 63% (LightGBM), 75% (SHAP), and 94% (Logistic Regression). Given that each method selects <1% of the total features, these overlap rates indicate substantial concordance beyond what would be expected from sparse selection alone.

### Drug target characterization

Analysis of drug target localization and protein translation patterns revealed that the selected targets tended to have fewer exons than average (see Supplementary Fig. 1E), although they were uniformly distributed across chromosomes (see Supplementary Fig. 1B). Genes with fewer exons are often involved in cell cycle regulation and other rapid-response processes, where reduced splicing complexity facilitates more efficient transcription and translation^17^.

**Fig. 2:**
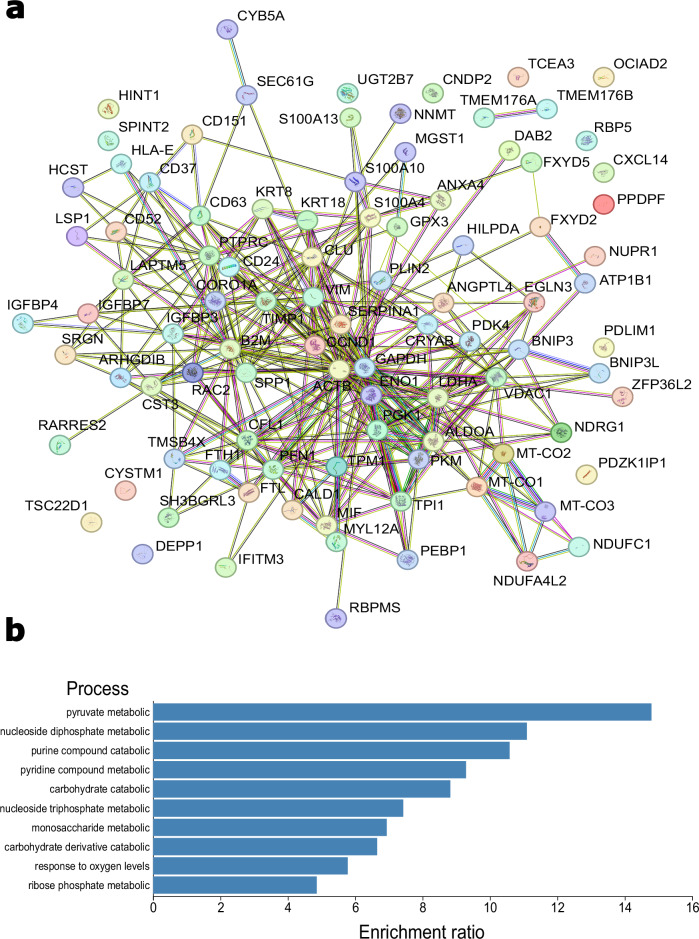
Functional and network characterization of tumor-specific drug targets. **a** Protein–protein interaction (PPI) network of the 96 selected drug targets, generated using STRING, demonstrating high connectivity and pathway convergence^18^. **b** Pathway enrichment analysis using WebGestalt reveals that drug targets are significantly enriched in pathways related to metabolism, oxygen sensing, and proliferation. Bar plot shows top enriched pathways ranked by enrichment ratio.

We next examined the biological functions of the drug targets. Using STRING, we constructed a protein–protein interaction (PPI) network, which revealed strong connectivity among the majority of targets (see Fig. 2a)^18^. Separate analyses of PPI networks for upregulated and downregulated genes further supported the functional interdependence of the identified targets (see Supplementary Fig. 1C, D).

We then explored the biological pathways associated with these proteins. The most significantly enriched pathways included carbohydrate metabolism, nucleoside turnover, and oxygen level responses (see Fig. 2b). The enrichment of carbohydrate metabolic pathways is consistent with the well-documented reliance of kidney tumors on glycolysis, characteristic of the Warburg effect^19,20^. Aberrant oxygen regulation, often mediated by VHL mutations, has also been extensively studied in ccRCC^21^. Similarly, altered nucleoside metabolism is frequently observed in cancer, where it supports the increased demand for nucleotides during rapid proliferation^22^.

To further validate the biological relevance of these targets, we examined their associations with key cancer hallmarks, including apoptosis, angiogenesis, and metabolic reprogramming. Most hallmarks were represented within the target gene set, with many individual targets contributing to multiple hallmark processes (see Supplementary Fig. 2A, B).

### Drug target signature validation

To validate the drug target signature, we utilised six external datasets originating from three different continents. The first dataset, collected in the United States, consists of scRNA-seq data from seven ccRCC tumor specimens and six benign human kidney samples^23^. The second, from China, includes scRNA-seq data from seven ccRCC tumors paired with five adjacent normal kidney samples^24^. The third, collected in Lithuania, contains scRNA-seq profiles from eight ccRCC tumors and nine matched healthy-adjacent kidney tissues^25^. In addition, we incorporated a fourth scRNA-seq dataset from the United States, comprising nine ccRCC bone metastasis samples^26^.

To provide broader population-level validation, we also analysed two bulk RNA-seq datasets. The TCGA ccRCC cohort includes 533 tumor samples and 73 normal kidney samples^27^. The second dataset, from Laskar et al., includes 503 tumor samples and 153 normal kidney samples^28^.

**Fig. 3:**
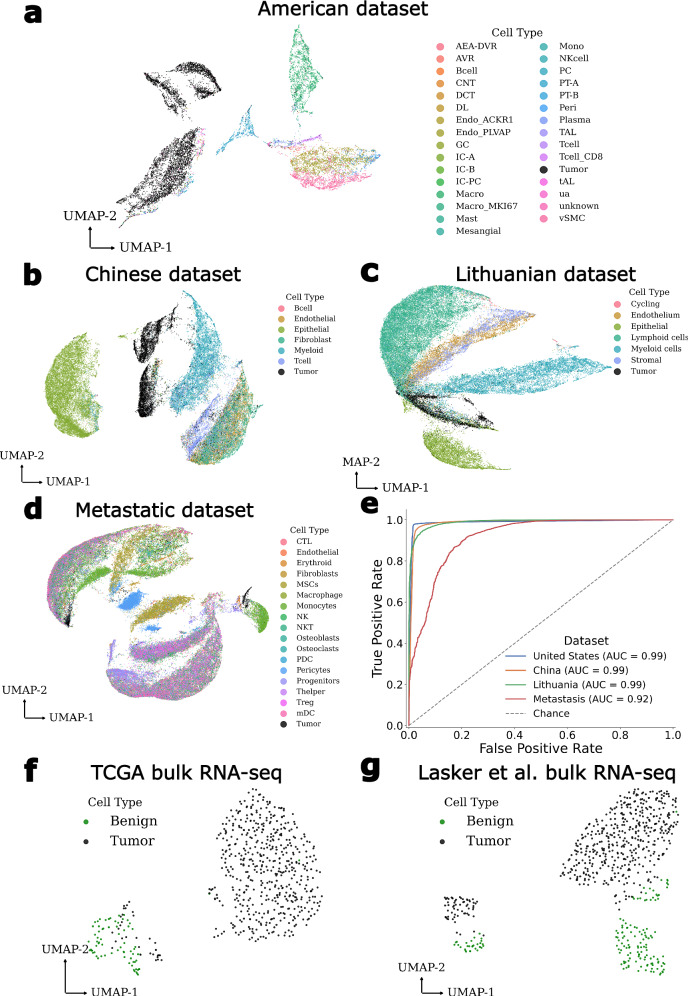
External validation confirms robustness of tumor-specific gene signature across independent datasets. **a**–**d** UMAP visualizations show effective tumor versus non-tumor separation using the 96-gene feature set (AUC ≥ 0.8) in four scRNA-seq datasets from the United States (Zhang et al.^23^) **a** China (Zhang et al.^24^) **b** Lithuania (Zvirblyte et al.^25^) (**c**) and ccRCC bone metastases (Mei et al.^26^) **d**, **e** ROC curves and AUC values confirm consistent classifier performance across diverse scRNA and bulk cohorts, with AUC values > 0.9 in all datasets. **f**, **g** UMAP plots of two bulk RNA-seq datasets (TCGA and Laskar et al.^28^) also demonstrate separation of tumor and normal samples despite cellular averaging.

We trained a LightGBM (LGBM) model to perform binary classification of tumor versus normal cells using the 96 drug targets across all cells in our training dataset These genes demonstrated strong discriminatory power, as visualized by UMAP embeddings that showed clear separation between tumor and non-tumor populations (Fig. 3a–d). The classifier was then applied to predict cell identities in the four external scRNA-seq datasets. The drug targets effectively distinguished cancerous from normal cells in all cases, with the classifier achieving high performance across datasets (see Fig. 3g). The AUC values were as follows: United States (0.99), China (0.99), Lithuania (0.99), and Metastasis (0.92). Additional performance metrics are provided in Supplementary Tables 2 and 3.

As expected, our model is not directly compatible with bulk RNA-seq data, due to fundamental differences in data modality. Unlike single-cell RNA-seq (scRNA-seq), which captures cell-type-specific expression profiles, bulk RNA-seq reflects the averaged gene expression across heterogeneous cell populations, potentially obscuring tumor-intrinsic signals. For instance, a gene that is upregulated in infiltrating lymphocytes but downregulated in tumor cells may still appear elevated in bulk tissue due to immune cell abundance.

Despite these limitations, UMAP visualizations revealed that the drug target gene signature could still separate tumor and normal samples in bulk RNA-seq datasets, demonstrating the robustness and generalizability of the selected features (see Fig. 3e, f). To further validate the performance of the proposed tree-based classifier and the selected features, we conducted 5-fold cross-validation on two independent bulk datasets, achieving high AUC values: TCGA-KIRC: 0.9853 ± 0.0204, Lasker: 0.9982 ± 0.0025. These results suggest that, while optimised for single-cell data, our feature set retains strong discriminatory power even in the bulk context.

### Hit identification

**Fig. 4:**
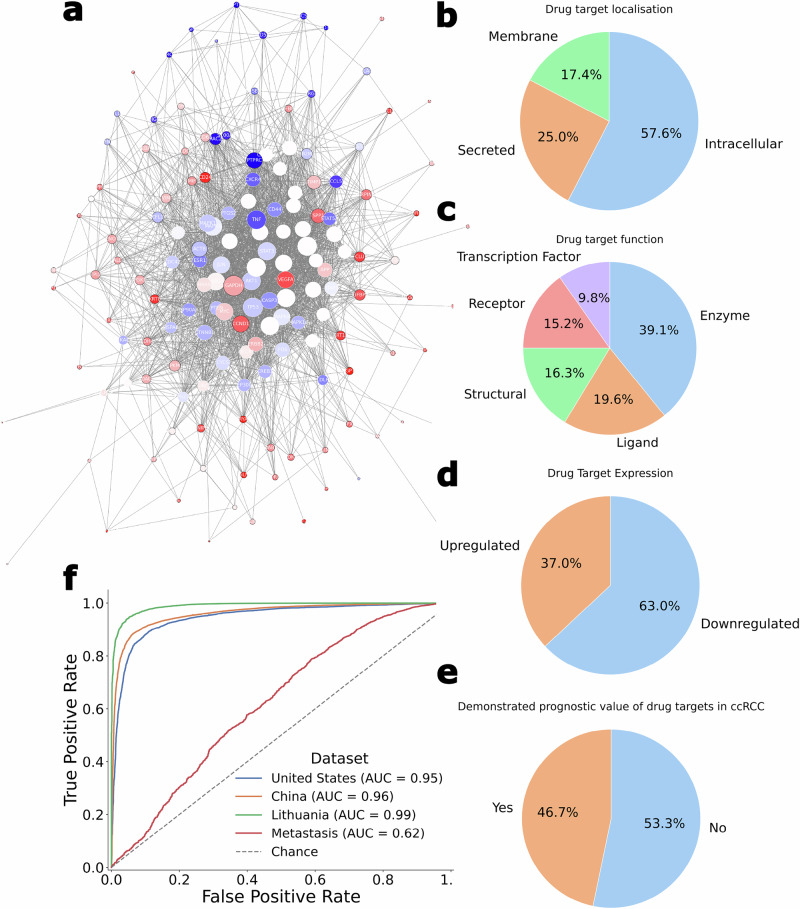
Network-guided refinement of drug targets reveals actionable therapeutic mechanisms. **a** Drug–target interaction network of the 92 key nodes, organised by subcellular localization: membrane, cytosol, and nucleus. Red = upregulated genes; blue = downregulated genes; white = unchanged. Size of the circles represent the size of the eigenvectors for each key node. **b**–**e** Summary of characteristics of identified key nodes: (**b**) Subcellular localisation; (**c**) Protein function (e.g., enzyme, receptor); (**d**) Direction of differential expression in tumor cells; (**e**) Prognostic significance in ccRCC based on Human Protein Atlas annotations^29^. **f** ROC curves showing classifier performance using the refined 16-gene target set across four external scRNA datasets.

Network analysis of the identified drug targets revealed 92 key nodes (see Supplementary File 3). Among these proteins, 57.6% were intracellular, 35% secreted, and 17.4% membrane-bound (see Fig. 4d–g). Functionally, the proteins were categorised as follows: 39.1% enzymes, 15.2% receptors, 19.6% ligands, 9.8% transcription factors, and 16.3% structural proteins. Expression analysis indicated that 63% were downregulated in tumor cells, while 37% were upregulated. Notably, 46.7% of the targets showed prognostic significance in ccRCC in the Human Cell Atlas^29^, and in many cases, ccRCC was the only cancer type in which these genes held prognostic value. Several central nodes, such as MYC, TP53, VEGFA, MAPK1, and CASP3, are well-established oncogenic drivers, further reinforcing the robustness of our network-based target selection (see Fig. 4a, b). The complete protein–protein interaction network is provided in Supplementary Fig. 3A.

To refine this list, we selected drug targets that were both identified through LightGBM-based feature selection and classified as key nodes in the network analysis, yielding the intersection of the 92 key nodes and 96 high-AUC genes. To ensure specificity, we excluded genes classified as common essentials by the DEPMAP CRISPR screen^30^. This refinement produced a final list of 23 drug targets (see Supplementary Table 4). To further refine the drug target set, we manually reviewed published literature for each gene. This step was critical, as some features identified through machine learning may reflect changes in the tumor microenvironment (e.g., immune infiltration) rather than intrinsic tumor biology. Supplementary Table 4 summarises the literature review. Seven genes were excluded due to unclear or contradictory links to tumor progression, often showing regulatory patterns inconsistent with prior studies. These were deemed more likely to reflect microenvironmental changes than true oncogenic drivers.

Using the remaining 16 targets, classification performance (AUC) remained high across the four scRNA-seq datasets: United States (0.94), China (0.96), Lithuania (0.91), and Metastasis (0.62) (see Fig. 4c). Additional metrics are presented in Supplementary Tables 5 and 6. UMAP visualization confirmed that the refined target set still effectively separates distinct cell populations (see Supplementary Fig. 6A–F).

We then performed drug proximity analysis on the 16 refined targets using both FDA-approved and non-FDA-approved compounds. Full results are presented in Supplementary Files 4 and 5. A proximity z-score cutoff of –2 was used to prioritize drug–target associations (see Supplementary Fig. 3B, C). Notably, three approved ccRCC therapies, Sorafenib, Sunitinib, and Pazopanib, were recovered by our pipeline, with Sorafenib and Sunitinib ranking among the top ten hits. Both Pazopanib and Sunitinib are first-line treatments for advanced ccRCC³², further validating our approach. We also identified sirolimus, a close analog of Everolimus, as a candidate mTOR inhibitor. Several additional VEGFR- and kinase-targeting drugs, such as Vandetanib, were also detected.

To prioritise translational potential, we focused on FDA-approved compounds, given their well-characterized safety profiles. This yielded 39 candidate drugs, which we categorised by mechanism of action (see Table 1). Several of these pathways have known anti-ccRCC activity. Among the previously underexplored mechanisms, we identified ABL1 inhibition, CDK4/6 inhibition, JAK inhibition, and non-VEGFR kinase inhibition as promising therapeutic strategies. Additionally, we identified the non-FDA-approved ROCK inhibitor Fasudil, currently used for subarachnoid hemorrhage, as a potential candidate for ccRCC. However, our downstream analysis focused on FDA-approved compounds due to their greater clinical tractability.

**Table 1:** Therapeutic mechanisms of FDA-approved drugs identified through network-based proximity analysis

Mechanism of action (MOA)	Drugs identified	MOA Comments
VEGFR inhibitors	Sunitinib, Sorafenib, Pazopanib, Vandetanib, Regorafenib	Already used in clinic
mTOR inhibitors	Sirolimus	Already used in clinic
JAK inhibitors	**Tofacitinib, Ruxolitinib**	Promising target
EGFR inhibitor	Erlotinib, Gefitinib, Trastuzumab, Afatinib	Successfully trailed in RCC^43^
MEK inhibitors	Trametinib	Successfully trailed in RCC^44^
CDK 4/6 inhibitors	**Ribociclib**	Promising target
Calcium metabolism modifiers	Amiodarone, Calcitriol	Benefits unclear
Hormone analogs	Stanozol, Nilutamide, Fulvestrant, Trilostane, Liotrix	Suggested as treatment for RCC but side-effects^45^
Dietary supplements	Zinc chloride	Benefits unclear
ABL1 inhibitors	**Bosutinib, Dasatinib, Nilotinib, Imatinib, Ponatinib**	Promising target
Antibiotic	Gentamicin	Nephrotoxic
Cytotoxic	Paclitaxel, Ingenol mebutate	Nephrotoxic
Clotting cascade modifier	Tranexemic acid, Tenecteplase	Benefits unclear
Glucocorticoids	Hydrocortisone acetate, Hydrocortisone	Successfully trailed in RCC in vitro^46^
Kinase inhibitors	**Fostamatinib**, Encorafenib, Zanubrutinib, Crizotinib	Promising target

Summary of repurposable FDA-approved drugs prioritised using network proximity to ccRCC-specific targets. Compounds are grouped by mechanism of action, with context provided on their relevance to clear cell renal cell carcinoma. Several pathways, such as VEGFR inhibition, mTOR inhibition, and multikinase targeting, are already used clinically, validating the approach. Others, including ABL1, CDK4/6, and JAK inhibition, represent novel strategies with demonstrated preclinical efficacy in this study. Agents with limited renal tolerability or unclear oncological relevance were deprioritised for downstream validation. Drugs indicated in bold very included in the preclinical validation.

### Preclinical drug validation

**Fig. 5:**
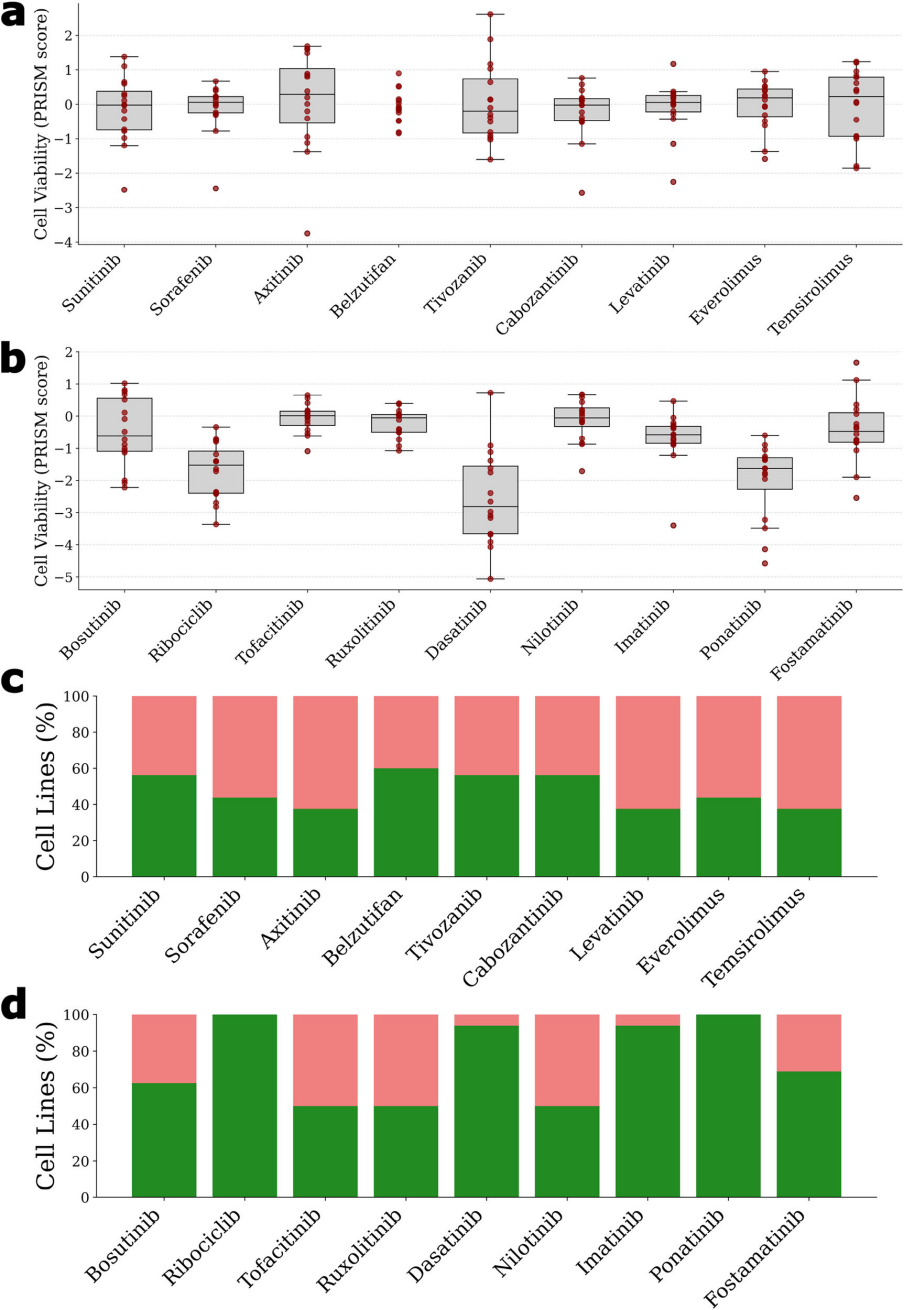
Predicted repurposed drugs outperform standard-of-care therapies in preclinical models. **a**-**b** Log₂ fold change in viability of ccRCC cell lines treated with approved drugs currently used in the clinic (**a**) versus top-ranked repurposed candidates identified in this study (**b**), using data from the PRISM 24Q2 screen. **c**, **d** Bar plots showing the proportion of ccRCC cell lines responsive to each compound, as determined by drug-induced viability reduction. Repurposed agents—including Ribociclib, Ponatinib, and Dasatinib—exhibited broader efficacy and more potent inhibition compared to all currently approved therapies. Green segments represent cell lines with reduced viability (inhibited), while pink segments denote non-responsive cell lines.

We assessed the efficacy of candidate compounds using the DEPMAP PRISM screen on 17 renal cell carcinoma (RCC) cell lines. As a baseline, we assessed nine FDA-approved therapies currently used to treat clear cell renal cell carcinoma (ccRCC). Consistent with clinical variability in treatment response, none of these agents reduced cell viability in >70% of the tested cell lines (see Fig. 5a and c). The most effective among them, the multikinase inhibitor Cabozantinib, achieved a mean log₂ viability reduction of –0.22, but failed to inhibit ~40% of RCC cell lines, consistent with clinical observations of resistance to current treatments.

In contrast, all nine of our proposed drug candidates demonstrated inhibitory effects comparable to or greater than those of standard-of-care therapies (see Fig. 5b and d). Notably, three predicted candidates, Ribociclib, Ponatinib, and Dasatinib, significantly reduced cell viability, with *p*-values of 0.00006, 0.00002, and 0.00008, respectively, compared to Cabozantinib. The corresponding log₂ fold changes in viability were –1.7, –2.0, and –2.5, respectively. Furthermore, both Ribociclib and Ponatinib inhibited viability in all tested cell lines, underscoring their potential as effective therapeutic options for RCC.

## Discussion

We present a systems-level computational drug discovery pipeline for clear cell renal cell carcinoma (ccRCC), combining single-cell transcriptomics, machine learning, and network analysis. While several individual components of our approach, such as tree-based feature selection, PPI network modeling, and network proximity scoring, are based on established methods, our contribution lies in the end-to-end integration of these steps into a coherent pipeline specifically tailored to ccRCC. In particular, our method: (i) converts cell-type-resolved scRNA-seq signals into a compact set of tumor-intrinsic target genes using robust feature selection, (ii) systematically refines these candidates using protein–protein interaction (PPI) network centrality and tumor-specific proximity constraints, and (iii) closes the loop with drug prioritization and validation using external compound response data (PRISM), ensuring translational relevance.

Previous computational repurposing efforts in kidney cancer have largely focused on individual genes^12^ or bulk RNA-derived signatures^22^. While some were validated in vitro or in xenografts, bulk RNA-seq lacks cell-type resolution, making it difficult to disentangle tumor-intrinsic signals from microenvironmental noise. A recent systems-level approach also relied on bulk expression data^13^ and did not recover known clinical agents, raising concerns about biological specificity. In contrast, our use of scRNA-seq enables cell-type-specific analysis, directly targeting malignant cell states rather than confounded tissue averages.

Experimental repurposing screens, such as high-throughput compound testing without molecular profiling^31^, remain important but are limited by cost, scalability, and lack of mechanistic insight. Our computational pipeline complements such efforts by prioritising compounds with predicted tumor-selective activity, informed by mechanistic multi-step filtering and validated using large-scale external datasets. Unlike prior computational studies, we demonstrate external validation of our prioritised drugs^12,13^, including recovery of clinically relevant compounds, supporting both the robustness and translational promise of our approach.

In our analysis, we identified several drug mechanisms consistent with current ccRCC treatments. These included VEGF receptor-targeting kinase inhibitors and multitarget kinase inhibitors. mTOR inhibitors were also recovered, although they ranked lower, aligning with their known modest response rates in ccRCC^9^. Among the 15 FDA-approved therapies for ccRCC, only 9 directly target the tumor when used in isolation; the remaining agents, such as immunomodulators and anti-VEGF antibodies, primarily act via the tumor microenvironment and thus would not be expected to show activity in our transcriptional or in vitro viability-based analysis. Within our top 40 predicted compounds, we successfully recovered both main tumor-targeted mechanisms: for VEGFR inhibition, we identified 3 out of the 9 clinically approved compounds; for mTOR inhibition, we did not recover the approved agents directly but did identify a close analog with a similar mechanism of action. These findings support the biological plausibility of our pipeline and highlight its capacity to recover known drivers of tumor progression, even in the absence of direct inclusion of clinical labels or prior knowledge.

Our analysis also revealed several compounds that are synthetic derivatives of endogenous hormones. Notably, the androgen Stanozolol and the antiandrogen Nilutamide, both used in prostate cancer and performance enhancement, were identified. While their therapeutic value in ccRCC remains unclear, these findings align with epidemiological data showing an almost two-fold increased risk of ccRCC among males^32^. In addition, the estrogen receptor antagonist Fulvestrant and the steroidogenesis inhibitor Trilostane, used in breast cancer, were also identified. Strogen is known to play a protective role in ccRCC development^33^. The identification of drugs with opposing hormonal effects is expected, as drug proximity scoring is undirected. Hormonal agents such as Medroxyprogesterone have previously been trailed in ccRCC but were largely discontinued due to side effects and limited efficacy^34^.

In the hit identification stage, we prioritised drug mechanisms that could be used alongside existing treatments to overcome resistance. We identified four underexplored therapeutic strategies for ccRCC using FDA-approved compounds: ABL1 inhibition, CDK4/6 inhibition, Janus kinase inhibition, and non-VEGFR kinase inhibition. We excluded drugs with broad systemic effects, such as corticosteroids, and filtered out nephrotoxic agents to prioritise compounds with safer profiles. Nine drugs targeting these mechanisms (see Table 1) were selected for preclinical evaluation, alongside nine standard-of-care therapies (see Fig. 5a, b). All selected candidates demonstrated comparable or superior inhibition of tumor cell viability. Notably, Ponatinib and Dasatinib (ABL1 inhibitors) and Ribociclib (a CDK4/6 inhibitor) significantly outperformed Cabozantinib, the most effective current treatment in our assay.

To explore combination potential, we evaluated pharmacological interactions using the DrugBank interactions database. Dasatinib inhibits CYP3A4, which metabolises frontline agents like Pazopanib, limiting its compatibility. However, both Ponatinib and Ribociclib are compatible with pazopanib, suggesting the possibility of a combination therapy involving three distinct mechanisms. Ponatinib, used in leukemia, primarily inhibits BCR–ABL but also targets KIT, RET, and Src kinases^35^. Ribociclib, a CDK4/6 inhibitor approved for breast cancer, has previously been proposed as a candidate for repurposing in other solid tumors, including RCC^36^.

We introduce a novel computational drug discovery pipeline leveraging single-cell RNA sequencing data and machine learning techniques. Our approach enables the identification of drug targets, which can be readily validated using classifiers on external datasets. The pipeline accurately identifies standard-of-care drug mechanisms to treat kidney cancer, demonstrating its accuracy. Additionally, we identify three highly efficacious new potential small molecules for ccRCC treatment with distinct mechanisms of action to current therapies. Due to high resistance to existing therapies in kidney cancer, these novel compounds show promise for improving clinical outcomes, especially in combination with current treatments.

This study has several limitations. Firstly, the identified compounds have not yet been evaluated in clinical settings for ccRCC. While all candidates have demonstrated safety and anti-cancer activity in other tumor types, their efficacy in ccRCC remains to be established. Although we validated our classifier on external datasets and supported drug prioritization using PRISM cell viability data, we acknowledge that classification performance alone does not guarantee therapeutic relevance of the selected targets. Furthermore, while the integration of network proximity with viability data offers mechanistic and phenotypic support, it does not by itself guarantee therapeutic efficacy. Additionally, while the systems-level drug analysis focuses on identifying classes of drug targets and overarching mechanisms of action, the downstream evaluation is performed at the level of individual compounds. Notably, compounds sharing the same primary mechanism can exhibit markedly different response rates, suggesting that secondary targets, off-target effects, or pharmacokinetic differences may influence efficacy.

Secondly, the proximity metric used in this study is undirected and may prioritise targets that are not directly druggable, necessitating manual curation. Thirdly, our pipeline primarily focuses on transcriptional differences between tumor and healthy cells, which may overlook tumor–microenvironment interactions. Future datasets with broader cell type representation from healthy kidney tissue could help identify additional targets, particularly those related to immune evasion or stromal signaling.

In addition to its findings in ccRCC, our study contributes a reusable framework for integrating single-cell transcriptomic data with interpretable machine learning and network-based drug discovery. Each module of the pipeline, from classifier training to feature selection and proximity scoring, is model-agnostic and adaptable, enabling application of the same structure to other cancer types or disease contexts. As single-cell datasets continue to expand across tumor systems, this framework offers a practical foundation for scalable, AI-driven therapeutic discovery, particularly in cancers limited by resistance or toxicity to current treatments.

## Methods

### Single cell RNA data preprocessing

The training dataset was obtained from a single center and comprised samples from ten ccRCC tumors, one oncocytoma, and one benign thick-walled cyst^15^. Cells were collected from normal kidney tissue, multiple tumor regions, and peripheral blood. Each cell was individually sequenced and subjected to quality control. Clustering and cell type annotation were performed using the *Seurat* package in R. The final dataset included over 270,000 cells.

For validation, we utilised six publicly available datasets:

1. Three single-cell RNA sequencing (scRNA-seq) datasets from tumor and adjacent tissues, originating from studies conducted in the United States^23^, China^24^, and Lithuania^25^.
2. One scRNA-seq dataset from the United States focusing on bone metastases and adjacent healthy tissue^26^.
3. Two bulk RNA-seq datasets^27,28^.

For each dataset, we used either the published cell annotations or re-annotated cells using the *Seurat* pipeline, applying marker genes provided in the respective studies. Although cell type labeling varied across datasets, this did not impact our analysis, as we focused primarily on distinguishing tumor cells from non-tumor cells. Donor annotations were available for all bulk RNA-seq samples. Genes absent in single-cell validation datasets were imputed as zero, while genes missing in the bulk RNA-seq datasets were excluded from the analysis, as they did not influence UMAP representations. An overview of the number of tumor and non-tumor samples for all the datasets used in this study is provided in Supplementary Table 1.

### Drug target identification

All subsequent data analysis was conducted using Python 3.12.9. For initial model exploration and classifier selection, we applied an 80:20 train–validation split stratified by cell type, for both multiclass and binary classification tasks. The final model was trained on the full dataset and evaluated on external test sets. Feature selection was performed using five-fold cross-validation, also stratified by cell type. As the binary tumor classification task was highly imbalanced, we applied the Synthetic Minority Oversampling Technique (SMOTE) to increase the representation of tumor cells in the training data with a positive to negative ratio used being 1:1 as we primarily interested in the difference between tumor and non-tumor^37^.

Both classification and feature selection were performed using the LightGBM algorithm from the Python package *lightgbm*^16^. Univariate feature selection was conducted using the SelectBySingleFeaturePerformance function from *feature-engine* library, in combination with a LightGBM model consisting of 1000 estimators, a maximum tree depth of 10, and 31 leaves, trained with a learning rate of 0.05 to minimise binary log loss. Although tree-based models can overfit when applied to single features in low signal-to-noise settings, we mitigated this risk by using cross-validation to compute the average area under the receiver operating characteristic curve (AUC) for each gene. The AUC cutoff of 0.8 was chosen to only include targets that have high predictive power. We also wanted to limit the number of genes identified to around 100 as otherwise the downstream analysis of the PPI networks becomes difficult as they become too dense to interpret.

For final classification, a more robust LightGBM model was used with 10,000 estimators and a learning rate of 0.01. To assess the stability and robustness of the selected features, we repeated the univariate analysis using logistic regression as an alternative estimator. In addition, we performed comparative feature selection by identifying the top 100 genes based on two complementary criteria: (i) SHAP values derived from the final LightGBM classifier, and (ii) the model-based feature importance scores. All UMAP visualizations were generated using the Python package *umap*, with the number of neighbors set to 15. The classifier’s performance on external datasets was assessed using accuracy, precision, recall, F1 score, and ROC-AUC to evaluate robustness across multiple performance metrics^38^.

### Network analysis

The identified drug targets were mapped to their corresponding proteins using BioMart. A protein–protein interaction (PPI) network was constructed using the *igraph* library, with interaction data obtained from the STRING database^39^, applying a confidence threshold of 0.425.

We previously developed a network-based drug repurposing pipeline to identify FDA-approved compounds targeting SARS-CoV-2 pathways^40^. In the present study, we applied the same network proximity analysis method, as described in Han et al., to assess the therapeutic relevance of key proteins identified within the ccRCC-specific interaction network. Specifically, given a set of key proteins KKK from our PPI network and a set of known drug targets TTT, we computed network proximity using the “closest” distance metric outlined by Guney et al.^41^, which has demonstrated effectiveness in drug–disease prediction tasks. The shortest distances between proteins were calculated using the *igraph* implementation of Dijkstra’s algorithm.

For each drug in the database the algorithm computes the shortest-path distance from every target to the closest disease protein (that mean is the observed distance d). To score proximity, the script resamples 1000 random target sets that match the original target-set degree profile. Each random draw produces a surrogate distance; the mean and standard deviation of those distances give *m* and *s*, and the proximity z-score is the usual standardised distance,

1 $$z=\frac{d-m}{s}.$$

Lower (more negative) *z* means the drug targets sit closer to the disease module than expected from degree-matched random targets. A binning strategy was used during random sampling to preserve the node degree distribution of the original network. Proteins with a proximity z-score below –2 (*p* < 0.01) were considered significantly proximal to known drug targets and prioritised as candidate therapeutic targets^41^. We evaluated node centrality using eigenvector centrality, degree centrality, betweenness centrality, and random-walk with restart/PageRank (RWR) on the original interaction graph.

To refine the target list, proteins classified as “common essentials” in the DEPMAP CRISPR 24Q4 screen were excluded^30^. The remaining candidates underwent manual curation to assess biological plausibility. Protein localization, function, and prognostic relevance in ccRCC were evaluated using The Human Protein Atlas^29^. The enrichment was calculated using the standard tools provided by WebGestalt^42^.

### Hit identification

We screened all FDA-approved drugs from DrugBank and non-FDA-approved compounds from PubChem. For each compound, network proximity to key proteins was calculated, and candidates with z-proximity scores below –2 were prioritised. The resulting drug list was then reviewed to ensure favorable safety profiles and known anti-cancer activity, while excluding compounds previously applied to renal cell carcinoma. To verify novelty, we searched ClinicalTrials.gov and PubMed using combinations of the drug name with the terms “renal carcinoma” or “kidney cancer”. Finally, drugs were categorised by their mechanisms of action to identify the most suitable compound for each therapeutic strategy.

### Preclinical drug validation

We assessed the efficacy of selected compounds using the PRISM Repurposing Public 24Q2 dataset provided by DEPMAP^30^. Our analysis focused on FDA-approved drugs predicted to reduce tumor cell viability based on their mechanisms of action. To maintain biological relevance, agents with broad systemic effects, such as immune checkpoint inhibitors and corticosteroids, were excluded. For baseline comparison, we considered only FDA-approved drugs for kidney cancer that act directly on tumor cells, yielding a set of 9 targeted agents primarily acting through VEGFR or mTOR inhibition. We excluded 6 additional approved compounds, such as immunotherapies and anti-angiogenic antibodies, due to their broad or microenvironment-focused mechanisms of action, which are not expected to show activity in transcriptional or in vitro viability-based analyses.

The DEPMAP screen included 20 renal cell carcinoma (RCC) cell lines, of which three were excluded due to their classification as non-ccRCC subtypes. Compounds were tested using the PRISM assay at a concentration of 2.5 μM for 5 days. Each compound was screened in triplicate, with every plate containing positive controls (bortezomib, 20 μM) and negative controls (DMSO) to ensure assay consistency. Differences in cell viability were evaluated using a two-tailed Mann–Whitney *U* test. A cell line was considered responsive if the log₂ fold change in viability was below zero.

For the final recommendation of compounds, we prioritised drug candidates with distinct primary mechanisms of action, targeting pathways or proteins not directly addressed by current ccRCC therapies. This was assessed manually to ensure that the predicted compounds could potentially act in complement to existing treatments, rather than redundantly. To further evaluate the compatibility and safety of potential combinations, we used the Drug Interaction Checker tool provided by DrugBank, enabling us to screen for known adverse interactions between our predicted drugs and currently approved therapies.

## Supplementary information

Supplementary Information

## Data Availability

All datasets used in this study are publicly available. The scRNA training data can be downloaded from Mendeley Data at: 10.17632/g67bkbnhhg.1. The scRNA validation data is accessible via the Gene Expression Omnibus (GEO) repository (https://www.ncbi.nlm.nih.gov/geo/) under accession numbers GSE156632, GSE159115, GSE242299, and GSE202813. The TCGA bulk RNA dataset can be downloaded from their website (https://xenabrowser.net/datapages/). The other bulk RNA-seq dataset is available in the GEO repository under accession number GSE242299. Since both the bulk RNA-seq and scRNA-seq datasets, as well as the preclinical cell viability data, were derived from previously published studies, no ethical concerns were raised.

## Code availability

The underlying code for this study is available on GitHub and can be accessed via this link https://github.com/Sr933/rcc.git. The code can also be requested from Silas Ruhrberg Estévez (sr933@cam.ac.uk).
